# Audiovisual integration of emotional signals from others' social interactions

**DOI:** 10.3389/fpsyg.2015.00611

**Published:** 2015-05-08

**Authors:** Lukasz Piwek, Frank Pollick, Karin Petrini

**Affiliations:** ^1^Behaviour Research Lab, Bristol Business School, University of the West of EnglandBristol, UK; ^2^School of Psychology, College of Science and Engineering, University of GlasgowGlasgow, UK; ^3^Department of Psychology, Faculty of Humanities & Social Sciences, University of BathBath, UK

**Keywords:** multisensory integration, social interactions, point-light displays, voice, happiness, anger

## Abstract

Audiovisual perception of emotions has been typically examined using displays of a solitary character (e.g., the face-voice and/or body-sound of one actor). However, in real life humans often face more complex multisensory social situations, involving more than one person. Here we ask if the audiovisual facilitation in emotion recognition previously found in simpler social situations extends to more complex and ecological situations. Stimuli consisting of the biological motion and voice of two interacting agents were used in two experiments. In Experiment 1, participants were presented with visual, auditory, auditory filtered/noisy, and audiovisual congruent and incongruent clips. We asked participants to judge whether the two agents were interacting happily or angrily. In Experiment 2, another group of participants repeated the same task, as in Experiment 1, while trying to ignore either the visual or the auditory information. The findings from both experiments indicate that when the reliability of the auditory cue was decreased participants weighted more the visual cue in their emotional judgments. This in turn translated in increased emotion recognition accuracy for the multisensory condition. Our findings thus point to a common mechanism of multisensory integration of emotional signals irrespective of social stimulus complexity.

## 1. Introduction

Perception of emotions is a multimodal event; by integrating signals from facial expressions, body movements, vocal prosody and other cues, we make emotional judgments about others. This multisensory integration of emotional expressions has been studied with faces and voices (de Gelder and Vroomen, 2000; Kreifelts et al., 2007; Collignon et al., 2008), body expression and faces (Meeren et al., 2005; Van den Stock et al., 2007), body expression with sound stimuli (Vines et al., 2006; Petrini et al., 2010, 2011), and body expressions and voices (Pichon et al., 2008; Stienen et al., 2011). A number of studies investigating the perception of emotions from facial expression and voices suggested strong bidirectional links between emotion detection processes in vision and audition (Massaro and Egan, 1996; de Gelder and Vroomen, 2000; Collignon et al., 2008; Jessen et al., 2012). For instance, de Gelder and Vroomen (2000) presented participants with static photographs of emotional faces combined with short vocal verbalizations, and found that participants emotional judgments reflected multisensory integration. When asked to identify the expression of a face, while ignoring a simultaneously heard voice, their judgments were nevertheless influenced by the tone of the voice, and vice versa. Similarly, Collignon et al. (2008) showed that participants were faster and more accurate to identify fearful and disgust expressions when they observed faces combined with voices than either faces or voices alone. This multisensory behavioral facilitation became particularly evident when the most reliable visual information was degraded, thus changing the participants weighting strategy (i.e., they weighted the auditory cue more when judging the expressed emotion). Only a small number of studies have examined how observers integrate signals from emotional body movement and voice, and results so far follow a similar pattern to studies of emotional faces and voices (Van den Stock et al., 2007).

These studies have examined perception of emotions involving faces, voices or body movement using single agent displays. However, a growing number of studies point to substantial differences between the social situations involving a single person compared to the situations involving two people interacting. Social interaction has been shown to change fundamental aspects of visual perception and recognition (Scherer, 2003; Shiffrar, 2011). For example, Neri et al. (2006) and Manera et al. (2011) demonstrated that observers can use information detected from one of the agents in the observed social interaction to predict the action or response from the other agent. Besides behavioral studies also neuroimaging studies (Centelles et al., 2011; Petrini et al., 2014) have examined which brain regions were recruited during the observation of two interacting agents. While the “mirror neuron” system and “mentalizing networks” are rarely concurrently active (Van Overwalle and Baetens, 2009), these studies found that both of these networks were needed to process the social intentions carried by the biological motion of the two humans interacting. This adds to the argument that observation and understanding of multiagent social interactions may involve a wider network of brain regions than that of a single agent social action.

We do not know however if these differences in behavioral and neural processing between multiagent and single agent social situations extend to multisensory recognition of emotions. Here we ask whether the multisensory facilitation in emotion recognition, reported by previous studies using single agent social displays (e.g., de Gelder and Vroomen, 2000; Collignon et al., 2008; Petrini et al., 2010, 2011), extends to multiagent social interactions. To this end we carried out two experiments, utilizing a paradigm frequently employed in studies of multisensory integration of emotional signals (e.g., de Gelder and Vroomen, 2000; Collignon et al., 2008; Petrini et al., 2010). In the both experiments we asked participants to recognize the emotion expressed (happiness or anger) in audiovisual, audio, and video clips of two agents interacting. In Experiment 1, we varied the reliability of the auditory information by using two different degrading methods (low -pass filtering and addition of brown noise), and the emotional congruency between visual and auditory cues. In Experiment 2, we also varied the level of relevance attributed to the two signals by asking participants to ignore one of the information while performing the task (e.g., to judge the visual emotion while ignoring the auditory emotion).

## 2. Materials and methods

### 2.1. Motion and voice capture of stimuli set

Motion capture took place at the University of Glasgow in the School of Psychology using 12 Vicon MXF40 cameras (Vicon, 2010) that offer online monitoring of 3D motion signals. The audio capture was done simultaneously using a custom-upgraded Vicon Analogue Card (Vicon, 2010) connected to amplifier with AKG D7S Supercardioid Dynamic Microphone, recording at 44.1 kHz and 24-bit sampling rate. Twelve repetitions of happy and angry interactions were recorded between eight pairs of actors (mean age of 26.12 years, ranging from 17 to 43 years). Actors were asked to interact exchanging one of two simple, single-sentence dialogues in each capture trial (e.g., Actor 1: “Where have you been?,” Actor 2: “I've just met with John”). A single capture trial lasted between 3–5 s. During the capture trial actors were positioned, one facing the other, at a distance specified by a marked position on the floor, approximately 1.3 m. This interpersonal distance varied between 1 and 1.6 m and it flexibly changed during the capture trials, depending on how much actors moved when interacting. However, at the beginning of each single capture trial actors were asked to come back to the start position marked on the floor.

To help actors convey angry and happy interactions they were given short and simple scenarios of the emotional situations and asked to imagine themselves in those situations. Actors were also instructed to recall their own past situations associated with the relevant emotional scenario to help them induce the emotion. The hypothetical scenarios were based on simple common situations (Scherer, 1986). Actors were given relative freedom in expressing the emotions during interactions (Clarke et al., 2005). They were encouraged to act naturally, but they were instructed to avoid touching each other and we were careful to give them only verbal instructions rather than performing actions ourselves (Clarke et al., 2005; Ma et al., 2006; Roether et al., 2009).

**Figure 1:**
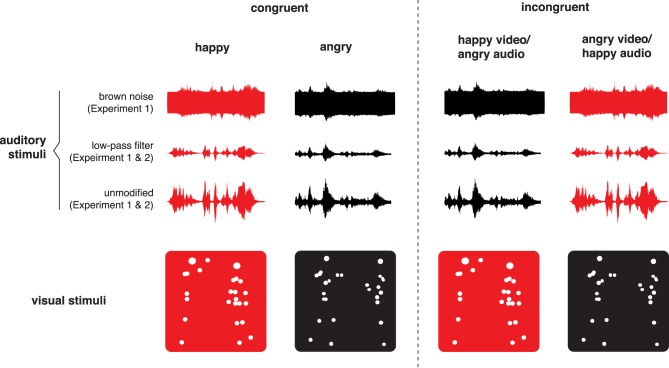
**Schematic explanation of creating bimodal incongruent stimuli**. Visual angry displays were combined with auditory happy displays, while visual happy were combined with auditory angry displays. Two types of auditory filtering were used in Experiment 1 (see Section 3.3), but only low-pass filtering was used in Experiment 2. For illustrative purposes, red represents happy displays and red-happy displays.

MATLAB 2010 (Mathworks, 2010) was used to convert captured movement into format useful for animation—as point-light displays. Point-light display (see Figure 1 for an example) is a method of representing movement separately from other cues like clothing or body shape and is one of the most common approaches in the study of human motion (Johansson, 1973). Point-light display contains little or no static spatial information and enables complex manipulation of different features such as temporal coordination (Bertenthal and Pinto, 1994) or position of points (Cutting, 1981; Verfaillie, 1993). We chose point-lights over full-body displays to avoid any emotional bias that could be associated with cues such as identity, clothing or body shape, and to make sure we are primarily looking at the effects of body movement with visual displays (Hill et al., 2003). Point-light displays also enable us to easily manipulate various parameters of displays (e.g., viewpoint, number of points), and therefore help us to “future proof” our stimuli set for other studies without the need to re-capture a new interactions.

To convert motion capture coordinates to point-light displays we used an approach similar to Dekeyser et al. (2002), Troje (2002), and Ma et al. (2006). Specifically, we computed the location of 15 virtual markers positioned at major joints of the body. The algorithm converted those 15 virtual markers from each actor into point-light displays (Pollick et al., 2001), generated as white dots on a black background from the side view. The algorithm exported point-light displays in the Audio Video Interleave (AVI) format with the frame rate of 60 fps.

Adobe Audition 3 (Adobe Systems, 2007) was used to post-process the dialogues. Every audio dialogue was first amplified by 10 dB and than a noise reduction was applied. All audio dialogues were than normalized to create a consistent level of amplitude, and to obtain the average volume of around 65 dB. Finally, each audio dialogue was exported as a Waveform Audio File Format (WAV) with a resolution of 44.1 kHz and 24-bit sampling rate.

The final stimulus was created using Adobe Premiere 1.5 (Adobe Systems, 2004) and consisted of 192 unique audio-visual clips (each clip was between 2500 and 3500 ms long) including 8 actor couples, 2 emotions (happy and angry) and 12 repetitions. Each unique clip was created in three modality formats: as point-lights (visual display), dialogue (auditory display) and a combination of point-lights with dialogues (audio-visual display). An example of angry and happy audio-visual clips can be viewed in Supplementary Movie.

### 2.2. Stimuli validation study

To examine whether observers could identify emotions conveyed in point-light displays and voice dialogues from created stimuli set, we conducted a stimuli validation study. Participant were presented with the displays as point-lights (visual group with 7 male and 8 female participants), voice dialogues (auditory group with 6 male and 7 female participants) or a combination of point-lights and dialogues (audio-visual group with 8 male and 7 female participants). Each group was presented with 192 displays described above. The reason for using a between-subject design was to avoid audio-visual facilitation, or carry-over effects, that could impact emotional identification when visual, auditory, and audio-visual displays are presented together in one set (Vines et al., 2006; Collignon et al., 2008). We also wanted to restrict presentation of every display to a single occasion to avoid effects of practice that can occur when participants see a repetition of a specific display (Heiman, 2002). The task was exactly the same for each group: after being presented with the display, participants were asked to identify whether interaction was happy or angry. Each display was presented only once and the order of all displays was randomized. The results provided us with average accuracy scores for each display we created in the stimuli set. Base on those results we selected a subset of eight angry and eight happy displays that were identified with 75% accuracy. However, by averaging across displays, we found that identification accuracy was higher in audio-visual (82%) and auditory-only (78%) groups than visual group (62%), indicating that the auditory information was more reliable than the visual. Hence, we decreased the reliability of the auditory stimuli to a level similar to the visual stimuli, as a greater increase in multisensory precision is obtained in situations for which the two sensory cues have a similar level of reliability (e.g., Ernst and Banks, 2002; Alais and Burr, 2004). To this end we used two methods frequently utilized in the literature: addition of brown noise to dialogues (Barnes and Allan, 1966; You et al., 2006; Hammerschmidt and Jürgens, 2007; Gardiner, 2009) and application of low-pass filter (Rogers et al., 1971; Frick, 1985; Scherer, 2003; Knoll et al., 2009). The use of both low-pass filtering and brown noise was guided by the principles of ecological validity—to choose a method of audio distortion that emulates real-life conditions. In this context, low-pass filtering made the voice dialogues sound like neighbors arguing behind a thick wall, or like the sounds heard when submerged in water; the words are unintelligible but the emotion behind the words is detectable. Accordingly, brown noise emulated real-life conditions such as listening to other peoples conversation during heavy rainfall. Examples of those filtering methods applied to happy and angry audio can be heard in Supplementary Movie.

## 3. Experiment 1

Experiment 1 examined whether participants were more accurate in recognizing the expressed emotions when presented with both visual and auditory signals than only visual or auditory. We used a similar procedure to the one applied by Collignon et al. (2008) and Petrini et al. (2010). Participants were asked to recognize angry and happy expressions either displayed aurally, visually or audio-visually, in a congruent (the same expressions in the two modalities) or incongruent way (different expressions in the two modalities).

### 3.1. Participants

A total of 31 participants were recruited for Experiment 1: 15 female and 16 male, with a mean age of 22 years, ranging from 17 to 34 years. All participants were English speakers and UK born. All reported normal hearing and normal or corrected-to-normal vision. All participants were naive to the purpose of the study and had no prior experience with point-light display movies or images. The study received ethical approval from the University of Glasgow's Faculty of Information and Mathematical Sciences Ethics Review Board and every participant signed a consent form.

### 3.2. Stimuli

The auditory stimuli consisted of unmodified voice dialogues, low-pass filtered (LPF) dialogues, and dialogues with brown noise applied to them. All dialogues were processed using Adobe Audition 3 (Adobe Systems, 2008). To create LPF versions of the dialogues, a filter with a 400 Hz cut-off was applied to the unmodified dialogues attenuating signals with frequencies higher than the cut-off frequency. It is sometimes called a high-cut filter, or treble cut filter in audio applications (MacCallum et al., 2011). To create noisy dialogues, brown noise was added to the unmodified clip. All clips were normalized to the same amplitude level of around 65 dB.

The visual stimuli were a side view, unmodified dyadic point-light displays, an example of which can be seen on Figure 1. The bimodal stimuli were obtained by combining corresponding point-light displays with voice dialogues. The matching could either be “congruent,” with the use of point-light displays and voice dialogues expressing the same emotion (e.g., angry point-lights/angry voices), or “incongruent,” with point-light displays and voice dialogues expressing different emotions (e.g., happy point-lights/angry voices). We created two incongruent versions of bimodal stimuli: point-light displays combined with unmodified voice dialogues, and point-light displays combined with dialogues filtered with brown noise or LPF. A schematic explanation of how bimodal incongruent stimuli were created is shown on Figure 1.

To summarize, the final stimuli set used in Experiment 1 consist of 112 stimuli with: 2 emotions (happy, angry), 7 stimulus types (visual, auditory unmodified, auditory filtered, bimodal congruent with unmodified dialogue, bimodal congruent with filtered dialogue, bimodal incongruent with unmodified dialogue, bimodal incongruent with filtered dialogue), and 8 actor pairs.

### 3.3. Design and procedure

Participants were tested in a dark room, with only a small lamp to illuminate the keyboard. They were seated approximately 65 cm from a 21″ Cathode Ray Tube (CRT) monitor with resolution of 1024 by 768 pixels, and 60 Hz refresh rate. Point-light displays subtended a maximum visual angle of approximately 8.5° in height and 6° in width. Voice dialogues were presented simultaneously with a white fixation cross shown during each display. Participants wore headphones (Beyer Dynamic DT Headphones), with an intensity at the sound source of 60 dB. We used Neurobehavioral Presentation 13.1 software (Neurobehavioral Systems, 2008) to present the displays and collect the responses. After each display, participants were asked to identify whether the presented interaction was happy or angry. They did so by pressing “H” for happy, or “A” for angry on the keyboard. Each display lasted between 2500–3500 ms and the next display was presented immediately after participants pressed the response key. Overall, participants were presented with a total of 336 displays that included three repetitions of all conditions randomly interleaved in 3 separate blocks of 112 stimuli.

Its important to note that in Experiment 1 auditory filtered stimuli were presented either with addition of brown noise (15 participants) or filtered with LPF (16 participants). We wanted to compare whether either of these two filtering methods was particularly better in filtering and decreasing reliability of auditory signal. We conducted two-sample *t*-tests on the averaged accuracy scores to establish whether there was a difference in correct discriminations when participants were presented with the auditory condition filtered with a low-pass filter rather than brown noise. Results showed that there was no significant difference in participants' performance between the two filtering methods (*t* = −0.42, *df* = 29, *p* = 0.68). Therefore, Experiment 1 included responses collated across two filtering methods as we found no differences between them.

### 3.4. Results

**Figure 2:**
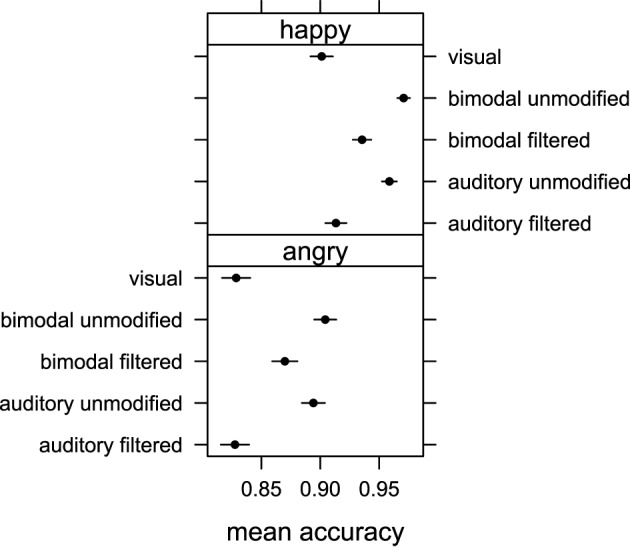
**Mean accuracy of emotion judgments obtained in Experiment 1 for auditory unmodified, auditory filtered, bimodal unmodified, bimodal filtered and visual stimuli conditions for happy and angry emotional expressions**. Error bars represent one standard error of the mean.

The averaged proportion of correct responses were submitted to a repeated measure ANOVA with “emotion” (happy and angry) and “stimuli” (visual, auditory unmodified, auditory filtered, bimodal congruent unmodified, and bimodal congruent filtered) as within factors. The ANOVA returned a main effect of “emotion” [*F*_(1, 29)_ = 13.81, *p* < 0.001, η^2^_*G*_ = 0.15]. Figure 2 clearly shows that participants were overall more accurate when judging happy rather than angry displays though the average recognition accuracy for the emotion expressed in the clips was far above the level of chance (50%). We also found a main effect of the factor ‘stimuli’ [*F*_(4, 116)_ = 20.46, *p* < 0.001, η^2^_*G*_ = 0.11] indicating that some stimuli conditions were judged more correctly than others. No interaction between factors “emotion” and “stimuli” [*F*_(4, 116)_ = 0.24, *p* = 0.91, η^2^_*G*_ = 0] was found, indicating that differences observed between various stimuli conditions were not influenced by emotional valence.

Pairwise comparison with correction for multiple testing showed that the emotion expressed in the visual displays was recognized less accurately than that expressed in the auditory unmodified (*p* < 0.001), bimodal unmodified (*p* < 0.001) and bimodal filtered (*p* < 0.001) displays. No difference in accuracy was found between visual and auditory filtered conditions (*p* = 0.56), and bimodal unmodified and auditory unmodified (*p* = 0.48). Finally, participants were more accurate in recognizing the correct emotion in the bimodal filtered condition than in either the auditory filtered condition (*p* < 0.001), or the visual condition (*p* < 0.001).

To analyze responses for incongruent bimodal stimuli we had to use a different approach, as there were no “correct” responses for this stimulus. We used the same approach of Collignon et al. (2008) and Petrini et al. (2010). We calculated a tendency to respond either “angry” or “happy” by subtracting the proportion of “happy” judgments from the proportion of “angry” judgments in the four incongruent stimulus conditions (happy point-light display/angry unmodified voice; happy point-light display/angry filtered voice; angry point-light display/happy unmodified voice; and angry point-light display/happy filtered voice). The index, which varied between -1 (subject always responded “happy”) and 1 (subject always responded “angry”) was then submitted to ANOVA with “auditory emotion” (happy or angry) and “auditory filtering” (filtered or unmodified) as within-subject factors.

**Figure 3:**
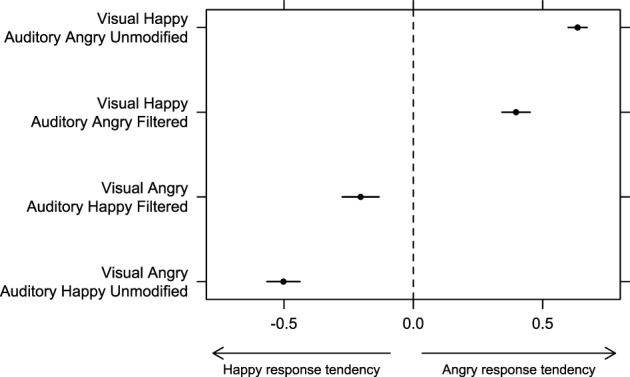
**Bias to respond either “happy” or “angry” in bimodal incongruent conditions was estimated by subtracting the proportion of “happy” responses from the proportion of “angry” responses in Experiment 1**. Error bars represent one standard error of the mean.

There was no significant effect of factor “auditory filtering” [*F*_(1, 30)_ = 1.49, *p* = 0.23, η^2^_*G*_ = 0], but we found a significant effect of factor “auditory emotion” [*F*_(1, 30)_ = 163.10, *p* < 0.001, η^2^_*G*_ = 0.65] as well as a significant interaction between factors “auditory emotion” and “auditory filtering” [*F*_(1, 30)_ = 86.07, *p* < 0.001, η^2^_*G*_ = 0.15]. Pairwise comparison with correction for multiple testing revealed that the index was significantly more positive with “visual happy/auditory angry unmodified” stimuli than with “visual happy/auditory angry filtered” (*p* < 0.01), and that the index was significantly more negative with “visual angry/auditory happy unmodified” stimuli than with “visual angry/auditory happy filtered” stimuli (*p* < 0.001). Figure 3 shows that for all bimodal incongruent combinations, participants' response were biased toward the auditory modality, but this tendency was weaker when filtering was present in the auditory signal. These results are consistent with the previous findings in showing a clear auditory dominance when no filtering or noise was applied, and a clear change in weighting strategy toward the visual information when the auditory reliability was decreased.

## 4. Experiment 2

In Experiment 2, we requested the participants to pay attention to only one modality at a time to ascertain whether any multimodal effects found in Experiments 1 were due to automatic processes and would not disappear when participants were asked to ignore one of the two modalities. The underlying idea was that if audio-visual integration operates in an automatic fashion, multisensory influence should occur even if the participants only focus their attention toward one single modality (de Gelder and Vroomen, 2000; Vroomen and de Gelder, 2000).

### 4.1. Participants

Sixteen participants were recruited for Experiment 2: 6 female and 10 male, with a mean age of 22.7 years, ranging from 18 to 36 years. All participants were English speakers and UK born. All reported normal hearing and normal or corrected-to-normal vision. All participants were naive to the purpose of the study and had no prior experience with point-light display movies or images. The study received ethical approval from the University of Glasgow's Faculty of Information and Mathematical Sciences Ethics Review Board and every participant signed a consent form.

### 4.2. Stimuli

The stimulus set used in Experiment 2 was exactly the same as in Experiments 1 (Section 3.2). As we didn't find a difference between two methods of auditory filtering in Experiment 1, we only used low-pass filter for audio filtering in Experiment 2 (see end of Section 3.3 for details).

### 4.3. Design and procedure

In Experiment 2 participants also performed an emotion recognition task but were explicitly asked to focus their attention on one sensory modality at a time, ignoring the other modality. As a result we introduced two separate blocks in Experiment 2: a visual and an auditory block. The visual block included 2 emotions (happy, angry), 5 stimulus types (visual, bimodal congruent with unmodified audio, bimodal congruent with filtered audio, bimodal incongruent with unmodified audio, bimodal incongruent with filtered audio), and 8 actor pairs. The auditory block included the same conditions of the visual blocks with only one difference; the auditory unimodal condition replaced the visual unimodal condition. Participants were presented with a total of 480 stimuli. Each block (i.e., auditory and visual) consisted of 240 stimuli, which included three repetitions of 80 stimulus conditions randomly interleaved within three separate blocks. Before starting to the visual block, participants were instructed to focus their attention on the visual displays and ignore the audio. In contrast, before starting to the auditory block, participants were instructed to focus their attention on the audio and ignore the visual displays. The order of visual and auditory blocks was counterbalanced across participants.

### 4.4. Results

**Figure 4:**
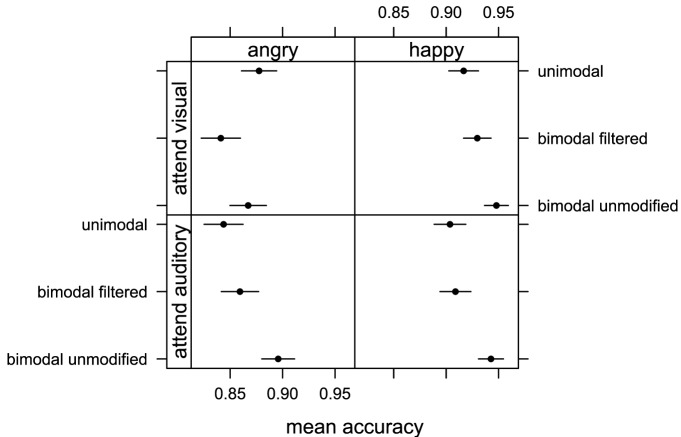
**Mean accuracy of emotion judgments obtained in Experiment 2 when participants attended visual information (top row with visual, bimodal unmodified, bimodal filtered stimuli) and auditory information (bottom row with auditory filtered, bimodal unmodified, bimodal filtered) for happy and angry emotional expressions**. Error bars represent one standard error of the mean.

The averaged proportion of correct responses were submitted to a repeated measure ANOVA with “emotion” (happy and angry), “attention” (attend vi-sual, attend auditory), and “stimuli” (unimodal, bimodal unmodified, and bimodal filtered) as within factors. We found a main effect of “emotion” [*F*_(1, 15)_ = 5.27, *p* < 0.05, η^2^_*G*_ = 0.10] and Figure 4 shows that participants were again more accurate when judging happy rather than angry displays. We also found a main effect of “stimuli” [*F*_(2, 30)_ = 6.35, *p* < 0.05, η^2^_*G*_ = 0.02] indicating that some stimulus conditions were judged with more accuracy than others. No interaction between ‘emotion’ and “attention” [*F*_(1, 15)_ = 0.16, *p* = 0.7, η^2^_*G*_ = 0]; “emotion” and “stimuli” [*F*_(2, 30)_ = 0.47, *p* = 0.63, η^2^_*G*_ = 0]; “attention” and “stimuli” [*F*_(2, 30)_ = 2.12, *p* = 0.14, η^2^_*G*_ = 0.01]; and “emotion,” “attention,” and “stimuli” [*F*_(2, 30)_ = 1.57, *p* = 0.23, η^2^_*G*_ = 0.01] was found. Pairwise comparison with correction for multiple testing showed that bimodal unmodified condition was judged more accurately than unimodal (*p* < 0.05) and bimodal filtered (*p* < 0.05) conditions. There was no difference between unimodal and bimodal filtered (*p* = 0.95). We found no significant effect of factor “attention” [*F*_(1, 15)_ = 0.11, *p* = 0.74, η^2^_*G*_ = 0] indicating that the level of accuracy for emotion recognition did not depend on the specific modality attended.

**Figure 5:**
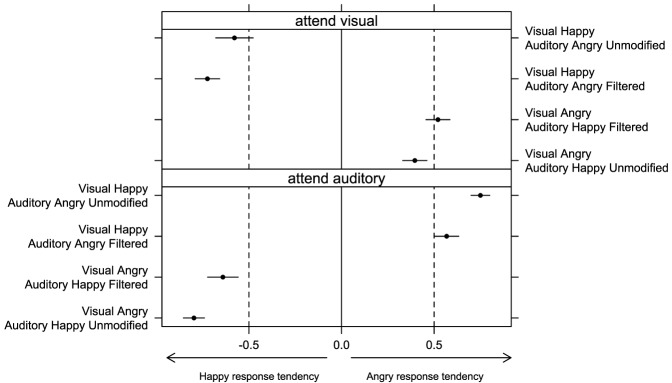
**Bias to respond either “happy” or “angry” in bimodal incongruent conditions was estimated by subtracting the proportion of “happy” responses from the proportion of “angry” responses in Experiment 2 (separately for conditions when participants attended visual or auditory signal)**. Error bars represent one standard error of the mean.

In Experiment 2, we again looked at the tendency to choose happy or angry emotion when observers were presented with incongruent displays. The index calculated for incongruent displays, which varied between −1 (subject always responded “happy”) and 1 (subject always responded “angry”), was analyzed by means of a Three-Way ANOVA with “auditory emotion” (happy or angry), “auditory filtering” (unmodified or filtered), and “attention” (visual or auditory) as within-subject factors. No significant effect of “attention” [*F*_(1, 15)_ = 1.93, *p* = 0.19, η^2^_*G*_ = 0.01] was found in line with the previous findings. Overall, Figure 5 shows that participants were biased toward the modality they attended—regardless of whether they attended auditory or visual signal. We found a significant effect of “auditory emotion” [*F*_(1, 15)_ = 7.11, *p* < 0.05, η^2^_*G*_ = 0.06] as well as a significant interaction between “auditory emotion” and “auditory filtering” [*F*_(1, 15)_ = 22.54, *p* < 0.001, η^2^_*G*_ = 0.07]. Figure 5 shows that the presence of auditory filtering weakened participants tendency to use auditory signal in their responses, but this effect was stronger with happy than angry audio (*p* < 0.05).

We also observed a significant interaction between “auditory emotion” and “attention” [*F*_(1, 15)_ = 245.45, *p* < 0.001, η^2^_*G*_ = 0.84]. Figure 5 shows that participants were biased toward the auditory information with the same extent for both happy and angry audio when they attended auditory rather than visual information. However, response tendency shifted more toward zero with happy audio than angry audio when visual signal was attended, which was not the case when auditory signal was attended.

Displays with happy auditory signal were shifted more toward the zero than with angry auditory signal when the visual was attended, but this is not the case when auditory was attended.

No other significant interaction was found.

## 5. Discussion

In the present study we ask whether the multisensory facilitation in emotion recognition, reported by previous studies using single agent social displays (e.g., de Gelder and Vroomen, 2000; Kreifelts et al., 2007; Collignon et al., 2008; Petrini et al., 2010), extends to multiagent social interactions. The results of both experiments consistently indicate that the auditory signal dominated the visual signal in the perception of emotions from social interactions. Participants were less accurate in discriminating emotions when making judgments on visual stimuli than on auditory stimuli. This result is in line with previous findings demonstrating that the auditory emotional information dominates the visual information in multisensory integration of emotional signals from body movements and sound (e.g., Vines et al., 2006; Petrini et al., 2010). However, degrading the auditory information so to match its level of reliability to that of the visual information changed the participants weighting of the two cues. The level of accuracy with which participants could recognize the emotion portrayed in the audio clips (when the auditory reliability was lower) was no better than that for the video clips. Integrating the two cues when the auditory was less reliable resulted in multisensory facilitation (i.e., participants were more accurate in recognizing the correct emotion when using both cues) as described by single agent studies (e.g., Collignon et al., 2008). Similarly, in both experiments we found that when participants judged the emotion in incongruent displays (e.g., happy visual information and angry auditory information), they shifted their responses toward the emotion represented by the visual signal if the auditory signal was less reliable. This supports earlier results by de Gelder and Vroomen (2000) and Collignon et al. (2008) that an incongruent combination of two signals would cause some disruption in the emotion interpretation of those signals, and a shift toward perceiving the emotion expressed by the most reliable information. The similarity between our findings and those using a single agent provides evidence for a common mechanism of multisensory integration of emotional signals irrespective of social stimulus complexity.

Our results also show an interesting difference in the way we interpret emotional signals from body movement and voice as compared to face and voice. Specifically, studies on the perception of emotions from face and voice show that observers make their judgments based mainly on faces rather than voices, although such dominance can shift depending on the visual and auditory reliability of the stimuli (Massaro and Egan, 1996; de Gelder and Vroomen, 2000; Collignon et al., 2008; Jessen et al., 2012). In contrast, our results suggest that auditory stimuli (voice) rather than visual stimuli (body movement) plays a particularly important role in the perception of emotional social interactions. Vines et al. (2006) and Petrini et al. (2010) show a similar patterns of results but with the musical sound dominating body expression when observers judged musical performance from those two cues. Petrini et al. (2010) highlight that making of music requires specific coupling between the performer and instrument, but the complexity of information in music sound is difficult to achieve with body expression. In short, body expression plays a “secondary” role as an accenting factor in the observation of musical performance. However, music is a special case since not only the majority of movements are constrained by the instrument, but those movements are also produced by a tool (the instrument) rather than coming from the body action *per se* (Petrini et al., 2010). Another possible explanation for the strong effect of voice found in our study is that we used point-light displays rather than full body displays. Reduced cue point-lights expressions could render visual signal less “informationally rich” when comparing to unmodified voice. Such argument is particularly valid when looking at the studies that used combination of static full body displays and voices (Stienen et al., 2011; Van den Stock et al., 2011). Specifically, those studies indicate that recognition performance for bodies and voices is on the similar level (i.e., visual signal is as reliable as auditory signal, as long as they are both congruent and unmodified).

Finally, its possible that the source of the sound from the dyadic point-light displays in our study is uncertain due to lack of conversational cues such as the mouth or face movements. A potential solution to those issues would be to increase the reliability of the visual signal by introducing full body displays (but with a blurred faces like in studies by Van den Stock et al. 2011) or Stienen et al. 2011), or to introduce conditions with only a single actor at the time so to specify the source of sound production.

In a separate argument, a broad literature on deception and non-verbal communication show a strong interrelation between body movement and voice. Ekman et al. (1976) found that measures of hand movements and voice were interrelated but changed incongruently when a person shifted from honest to deceptive expressions. Specifically, the amount of symbolic hand movements decreased in deception, while pitch variance into high tones increased with deception, making the voice more accessible as cue as well as creating a discrepancy between voice and body movement. Moreover, studies on body movement and speech rhythm in social conversation clearly show that speakers tend to use their body movement to highlight specific aspects of their spoken messages (Dittmann and Llewellyn, 1969). Movement output and speech output were found to be quite closely correlated (Boomer, 1963). Renneker (1963, p. 155) described what he called “speech-accompanying gestures,” which “seek to complement, modify, and dramatize the meanings of words,” Freedman and Hoffman (1967) separated what they called “punctuating movements” from other speech-related movements. It is possible that, in a conversational context, body movements play an accenting function to the voice—a claim also supported and suggested by Ekman (1965) regarding non-verbal behavior in general. This claim is further supported by brain imaging studies. For instance, Hubbard et al. (2009) found that non-primary auditory cortex showed greater activity when speech was accompanied by “beat” gesture than when speech was presented alone. Hubbard et al. (2009) results pointed toward a common neural substrate for processing speech and gesture, likely reflecting their joint communicative role in social interactions.

Considering our results on the emotional identification, we found that happy interactions were repeatedly identified more accurately than angry interactions in both experiments. The accuracy of recognition between angry and happy affect has long been a point of debate between researchers. A number of studies have shown that observers were better at identifying angry rather than happy emotional expressions when listening to voices (Scherer, 1986), viewing faces (Massaro and Egan, 1996; Fox et al., 2000; Knyazev et al., 2009), watching the actions of a single actor (Pollick et al., 2001) or watching interactions between two actors (Clarke et al., 2005). Several studies also argue that detection of anger serves as an evolutionary indicator of threat (Pichon et al., 2008), and specific brain areas such as the amygdala are tuned to detect angry actions from body movement (de Gelder, 2006). However, others found similar results to ours highlighting that happy expression is a highly salient social signal. For example, Dittrich et al. (1996) showed that happy displays of point-light dancers were identified more accurately compared to angry displays. Belin et al. (2008) created and experimentally validated a dataset of non-verbal affect bursts showing that vocal expressions of happiness were better recognized than anger. Johnstone et al. (2006) found that greater activation to happy vs. angry vocal expressions in amygdala and insula regions when explicitly attending to these expressions. In such context, our study adds further evidence that happy expressions from movement and voice are potentially more salient social signals when compared to anger.

In conclusion, we found that the auditory signal dominated the visual signal in the perception of emotions from social interactions, but only to the extent of auditory signals' reliability. When reliability of auditory signal was degraded, participants weighted visual cues more in their judgments, which followed pattern of results similar to de Gelder and Vroomen (2000), Collignon et al. (2008), and Petrini et al. (2010). Similarly, when participants watched emotionally mismatched bimodal displays, filtering auditory signal increased the weighting of visual cue. Our results suggest that when identifying emotions from complex social stimuli, we use similar mechanism of multimodal integration as with simple social stimuli.

## Author contributions

Conception and design of the work: LP, KP, FP. Stimuli capture, processing and production: LP, FP. Acquisition of the data: LP. Analysis of the data: LP, KP. Wrote the paper: LP, KP, FP.

### Conflict of interest statement

The authors declare that the research was conducted in the absence of any commercial or financial relationships that could be construed as a potential conflict of interest.
